# Roary: rapid large-scale prokaryote pan genome analysis

**DOI:** 10.1093/bioinformatics/btv421

**Published:** 2015-07-20

**Authors:** Andrew J. Page, Carla A. Cummins, Martin Hunt, Vanessa K. Wong, Sandra Reuter, Matthew T.G. Holden, Maria Fookes, Daniel Falush, Jacqueline A. Keane, Julian Parkhill

**Affiliations:** ^1^Pathogen Genomics, The Wellcome Trust Sanger Institute, Wellcome Trust Genome Campus, Hinxton, Cambridge,; ^2^Department of Medicine, University of Cambridge, Cambridge,; ^3^School of Medicine, University of St. Andrews, North Haugh, St Andrews and; ^4^College of Medicine, Swansea University, Swansea, UK

## Abstract

**Summary:** A typical prokaryote population sequencing study can now consist of hundreds or thousands of isolates. Interrogating these datasets can provide detailed insights into the genetic structure of prokaryotic genomes. We introduce Roary, a tool that rapidly builds large-scale pan genomes, identifying the core and accessory genes. Roary makes construction of the pan genome of thousands of prokaryote samples possible on a standard desktop without compromising on the accuracy of results. Using a single CPU Roary can produce a pan genome consisting of 1000 isolates in 4.5 hours using 13 GB of RAM, with further speedups possible using multiple processors.

**Availability and implementation:** Roary is implemented in Perl and is freely available under an open source GPLv3 license from http://sanger-pathogens.github.io/Roary

**Contact:** roary@sanger.ac.uk

**Supplementary information:** Supplementary data are available at *Bioinformatics* online.

## 1 Introduction

The term microbial pan genome was first used in 2005 (Medini *et al.*, 2005) to describe the union of genes shared by genomes of interest (Vernikos *et al.*, 2014). Since then, availability of microbial sequencing data has grown exponentially. Aligning whole-genome-sequenced isolates to a single reference genome can fail to incorporate non-reference sequences. By using *de novo* assemblies, non-reference sequences can also be analyzed. Microbial organisms can rapidly acquire genes from other organisms that can increase virulence or promote antimicrobial drug resistance (Medini *et al.*, 2005). Gaining a better picture of the conserved genes of an organism, and the accessory genome, can lead to a better understanding of key processes such as selection and evolution.

The construction of a pan genome is NP-hard (Nguyen *et al.*, 2014) with additional difficulties from real data due to contamination, fragmented assemblies and poor annotation. Therefore, any approach must employ heuristics to produce a pan genome (reviewed in Vernikos *et al.* 2014). The most complete standalone pan genome tools are PanOCT (Fouts *et al.*, 2012), which uses a conserved gene neighborhood in addition to homology to accurately place proteins into orthologous clusters; LS-BSR (Sahl *et al.*, 2014) which uses a preclustering step before running BLAST to rapidly assign genes to families and PGAP which takes annotated assemblies, performs an all-against-all BLAST, clusters the results and produces a pan genome (Zhao *et al.*, 2012).

PanOCT and PGAP require an all-against-all comparison using BLAST, with the running time growing approximately quadratically with the size of input data and are computationally infeasible with large datasets. They also have quadratic memory requirements, quickly exceeding the RAM available in high performance servers for large datasets. LS-BSR introduces a pre-clustering step that makes it an order of magnitude faster than PGAP; however, it is less sensitive (Sahl *et al.*, 2014). We have developed a method to generate the pan genome of a set of related prokaryotic isolates. It works with thousands of isolates in a computationally feasible time, beginning with annotated fragmented *de novo* assemblies. We address the computational issues by performing a rapid clustering of highly similar sequences, which can reduce the running time of BLAST substantially, and carefully manage RAM usage so that it increases linearly, both of which make it possible to analyze datasets with thousands of samples using commonly available computing hardware.

## 2 Description

The input to Roary is one annotated assembly per sample in GFF3 format (Stein, 2013), such as that produced by Prokka (Seemann, 2014), where all samples are from the same species. Coding regions are extracted from the input and converted to protein sequences, filtered to remove partial sequences and iteratively pre-clustered with CD-HIT (Fu *et al.*, 2012). This results in a substantially reduced set of protein sequences. An all-against-all comparison is performed with BLASTP on the reduced sequences with a user defined percentage sequence identity (default 95%). Sequences are then clustered with MCL (Enright *et al.*, 2002), and finally, the pre-clustering results from CD-HIT are merged together with the results of MCL. Using conserved gene neighborhood information, homologous groups containing paralogs are split into groups of true orthologs. A graph is constructed of the relationships of the clusters based on the order of occurrence in the input sequences, allowing for the clusters to be ordered and thus providing context for each gene. Isolates are clustered based on gene presence in the accessory genome, with the contribution of isolates to the graph weighted by cluster size. A suite of command line tools is provided to interrogate the dataset providing union, intersection and complement. Full details of the method and outputs are provided in the Supplementary Material.

## 3 Results

We evaluated the accuracy, running time and memory usage of Roary against three similar standalone pan genome applications. In each case, we performed the analysis using a single processor (AMD Opteron 6272) and provided 60 GB of RAM. We constructed a simulated dataset based on *Salmonella enterica* serovar Typhi (*S.typhi*) CT18 (acc. no. AL513382), allowing us to accurately assess the quality of the clustering. We created 12 genomes with 994 identical core genes and 23 accessory genes in varying combinations. All the applications created clusters that are within 1% of the expected results, with Roary correctly building all genes as shown in Table 1. The overlap of the clusters is virtually identical in all applications except LS-BSR, which over clusters in 2% of cases.

**Table 1.** Accuracy of each pan genome application on a dataset of simulated data

	Core genes	Total genes	Incorrect split	Incorrect merge
Expected	994	1017	0	0
PGAP	991	1012	0	4
PanOCT	993	1015	1	1
LS-BSR	974	994	0	23
Roary	994	1017	0	0

**Fig. 1.**
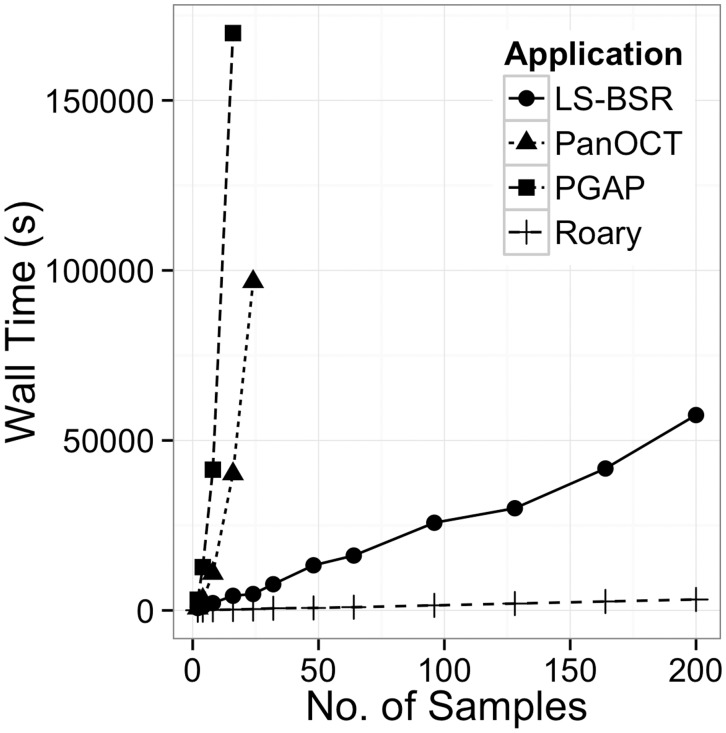
Effect of dataset size on the wall time of multiple applications. Only analysis that completed within 2 days and 60 GB of RAM is shown

In addition, a set of 1000 real annotated assemblies of *S.typhi* genomes was used. Subsets of the data were provided to each application, and the running time and memory usage were noted. The running time of PGAP and PanOCT increases substantially, making only small datasets computationally feasible (Fig. 1 and Supplementary Figs S1–S8). Roary scales consistently as more samples are added (Supplementary Figs S1–S8) and has been shown to work on a dataset of 1000 isolates as shown in Table 2. The memory usage of PGAP and PanOCT also increases rapidly as more samples are added, quickly exceeding 60 GB for even small datasets. The memory usage of Roary scales consistently as more samples are added, making it feasible to process large datasets on a standard desktop computer within a few hours. We conducted similar experiments with more diverse datasets including *Streptococcus* *pneumonia*, *Staphylococcus* *aureus* and *Yersinia* *enterocolitica* and the results exhibit similar speed-ups as shown in Supplementary Figures S7 and S8. The performance in a multi-processor environment is shown in Supplementary Figs S11 and S12, with Roary achieving a speedup of 3.7X using 8 CPUs and GNU Parallel (Tang, 2011).

**Table 2.** Comparison of pan genome applications using real *S.typhi* data (ERP001718)

Samples	Software	Core^a^	Total	RAM (mb)	Wall time (s)
8	PGAP	4545	4929	569	41 397
	PanOCT	4544	4936	663	1457
	LS-BSR	4476	4816	270	2585
	Roary	4459	4871	156	44
24	PGAP	—	—	—	—
	PanOCT	4522	4991	5313	96 093
	LS-BSR	4451	4843	554	7807
	Roary	4436	4941	444	382
1000	PGAP	—	—	—	—
	PanOCT	—	—	—	—
	LS-BSR	4272	7265	17 413	345 019
	Roary	4016	9201	13 752	15 465

^a^Core is defined as a gene being in at least 99% of samples, which allows for some assembly errors in very large datasets. Where there are no results, the applications failed to complete within 5 days or used more than 60 GB of RAM. The first column is the number of unique *S.typhi* genomes in the input set with a mean of 54 contigs over all 1000 assemblies.

## 4 Discussion

We have shown that Roary can construct the pan genomes of large collections of bacterial genomes using a desktop computer, where it was not previously computationally possible with other methods. Further speedups in running time are possible by providing more processors to Roary. On simulated data, Roary is the only application to correctly identify all clusters. This increased accuracy comes from using the context provided by conserved gene neighborhood information. Roary scales well on large real datasets, identifying large numbers of core genes, even in the presence of a varied open pan genome.

## Supplementary Material

Supplementary Data
