# Supplementary material for: Roary: rapid large-scale prokaryote pan genome analysis
Source: Bioinformatics. 2015 Jul 20;31(22):3691–3. doi: 10.1093/bioinformatics/btv421 (PMC4817141; doi:10.1093/bioinformatics/btv421)
Supplement: Supplementary Data [file supp_31_22_3691__index.html]

Roary: Rapid large-scale prokaryote pan genome analysis — Roary: rapid large-scale prokaryote pan genome analysis — Roary: rapid large-scale prokaryote pan genome analysis — Supplementary Data 

# Roary: rapid large-scale prokaryote pan genome analysis

## Supplementary Data

files

- Supplementary Data - xls file
- Supplementary Data - pdf file
