# Supplementary material for: Roary: rapid large-scale prokaryote pan genome analysis
Source: Bioinformatics. 2015 Jul 20;31(22):3691–3. doi: 10.1093/bioinformatics/btv421 (PMC4817141; doi:10.1093/bioinformatics/btv421)
Supplement: Supplementary Data [file supp_btv421_Roary_supplementary_material.pdf]

Roary: Supplementary Material

- 1 Performance Evaluation
  - 1.1 Comparison to other methods
  - 1.2 Clusters identified
  - 1.3 Scaling in a multiprocessor environment
- 2 Method
  - 2.1 Input
  - 2.2 Method description
  - 2.3 Output
- 3 Output files
  - 3.1 Core gene alignment
  - 3.2 Multifasta files of each gene
  - 3.3 Pan Genome Reference FASTA
- 4 Command line tools
  - 4.1 Querying the pan genome
    - 4.1.1 Difference between sets of isolates
    - 4.1.2 Create multi-FASTA files for a list of genes
    - 4.1.3 Union (pan genome)
    - 4.1.4 Intersection (core genes)
    - 4.1.5 Complement (accessory genes)
- 5 References

1 Performance Evaluation

1.1 Comparison to other methods

For the performance evaluation we used a set of 200 *Salmonella enterica serovar Typhi (S. typhi)* samples collected between 2007 and 2012. Additionally we used datasets of from *Streptococcus pneumoniae (S. pneumoniae)*, *Staphylococcus aureus (S. aureus)* and *Yersinia enterocolitica (Y. enterocolitica)*. Each of these datasets contained 100 samples and have a varying degree of diversity. All samples were sequenced on the Illumina 2000 and 2500 platforms, *de novo* assembled using Velvet Optimiser, and annotated using Prokka. Accession numbers for the sequence data and annotated assemblies used in our analysis are provided in Supplementary Material file 2.

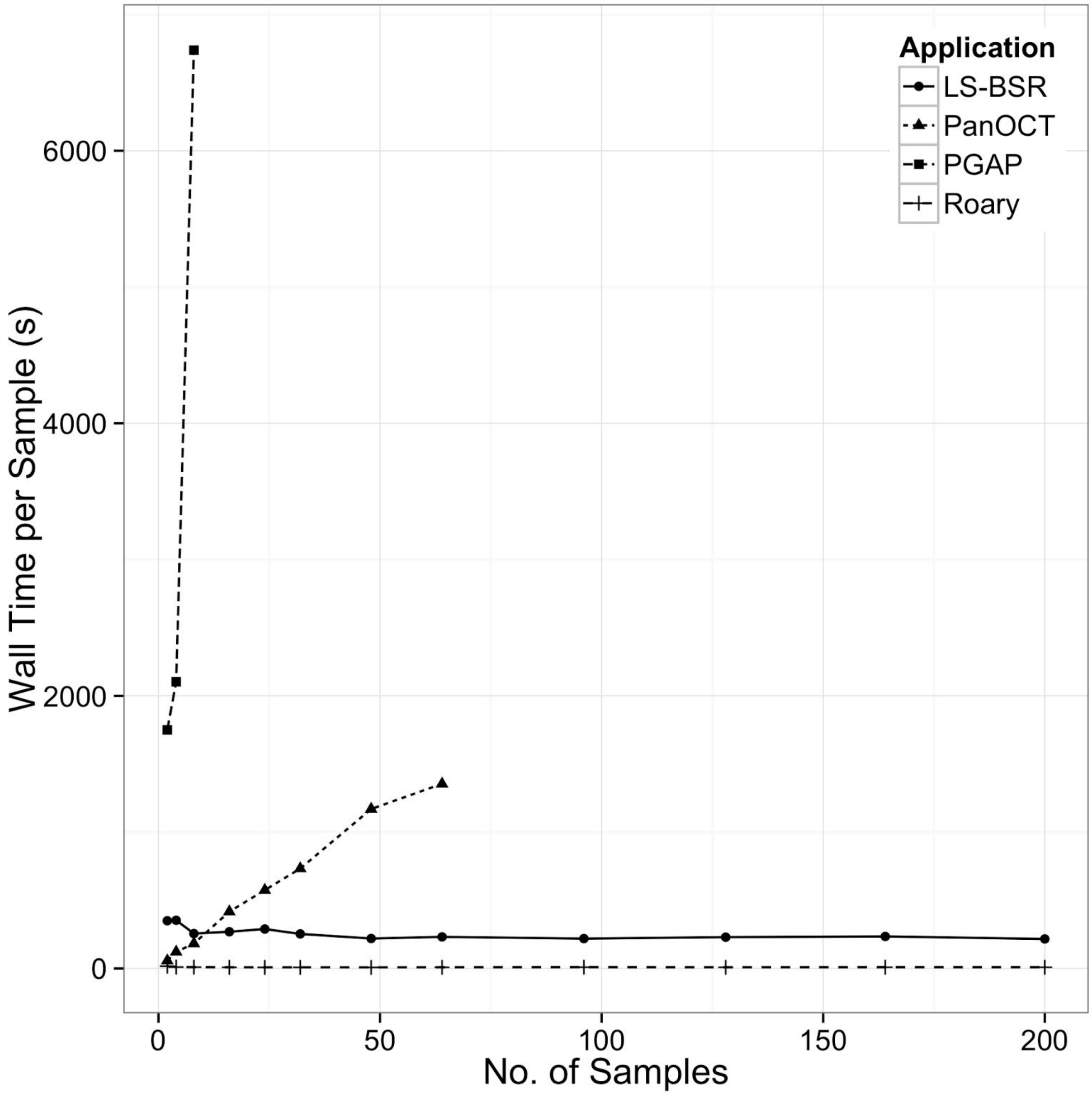

Sup. Fig. 1: The wall time in seconds for processing *S. typhi* samples with increasing sizes of data sets using 8 CPUs. Roary has a constant processing time for samples, regardless of the size of the number of samples, PanOCT scales linearly and PGAP scales exponentially. PanOCT and PGAP failed to run for large data sets because they exceeded 48 hours or 60GB of RAM.

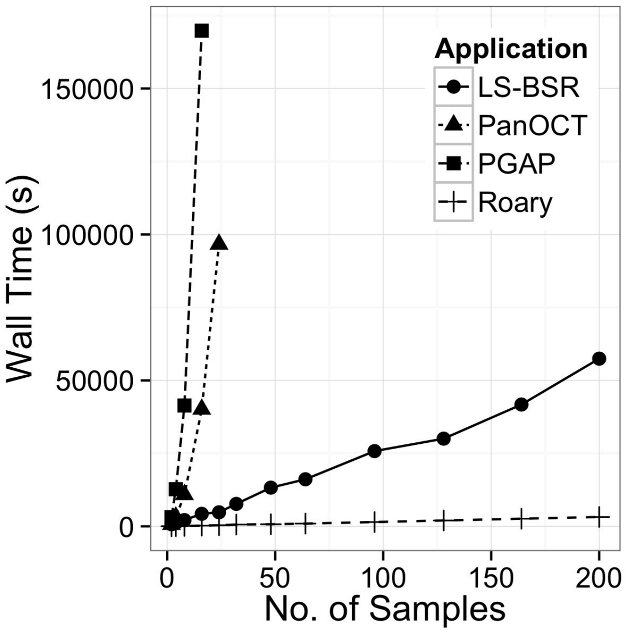

Sup. Fig 2: Running time in seconds with increasing numbers of samples using a single CPU on a dataset of *S. typhi.* Roary increases linearly. The other applications have such a high running time that they can only realistically be run on small datasets.

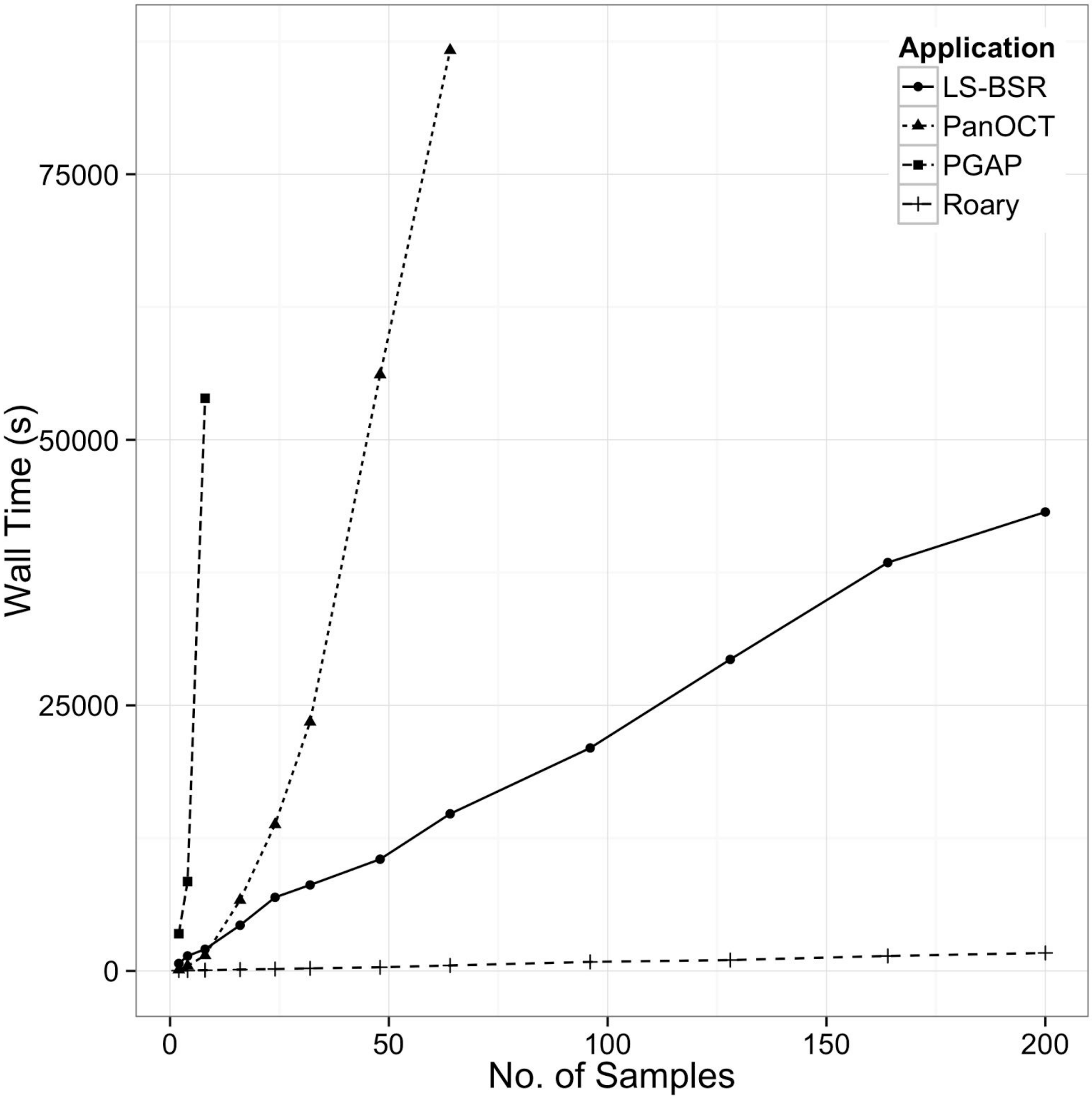

Sup. Fig. 3: Running time with 8 CPUs and increasing numbers of *S. typhi* samples. With Roary adding more CPUs reduces the running time, making larger dataset more feasible.

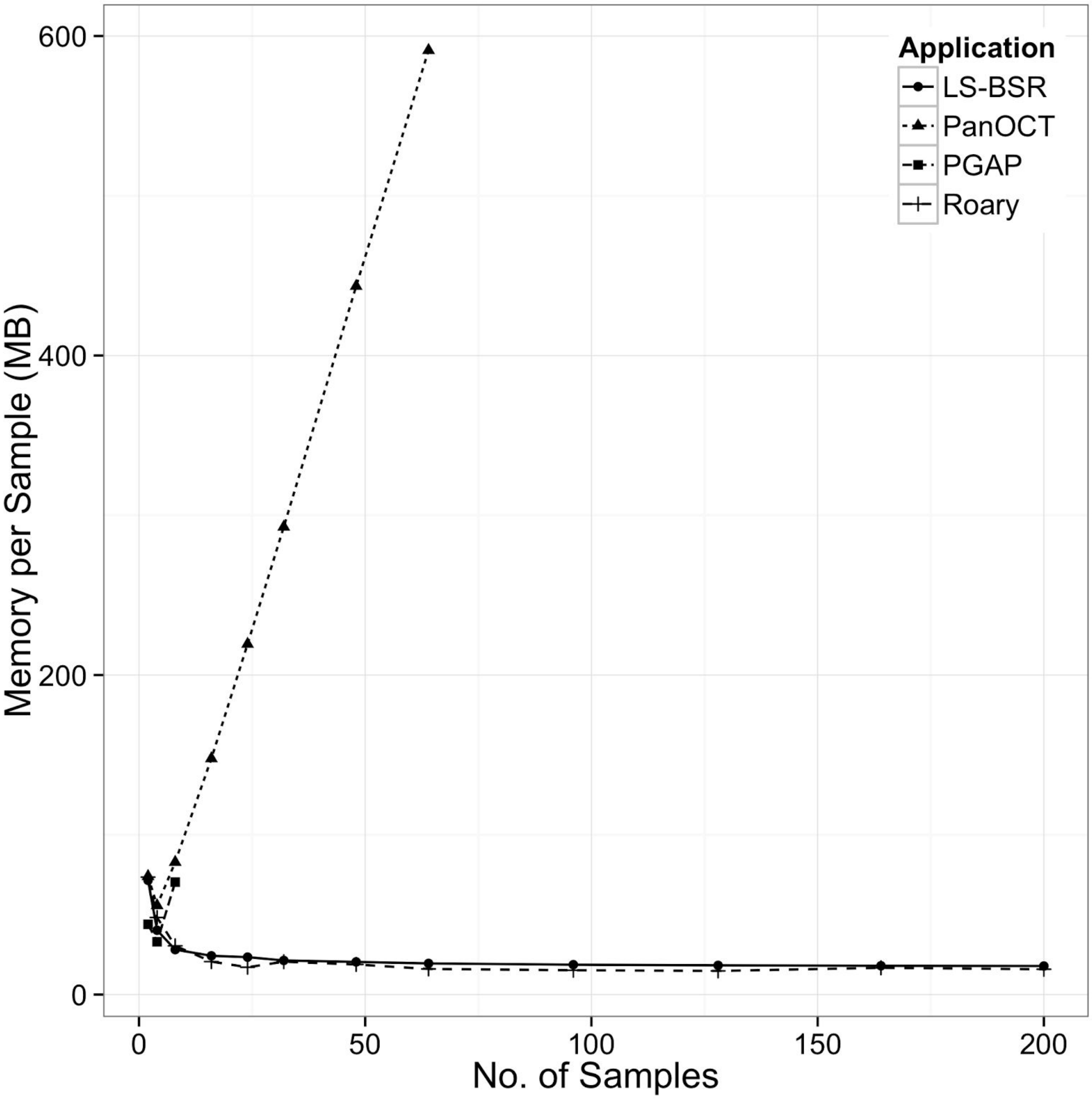

Sup. Fig. 4: Memory used per sample in MB with increasing numbers of *S. typhi* samples, using 8 CPUs. Roary quickly stabilises to a constant level. This is related to the size of the whole pan genome, so if the total number of genes is stable, then so is the memory requirement. PanOCT has an exponential memory requirement. There are not enough data points to draw conclusions about PGAP. PanOCT and PGAP failed to run for large data sets because they exceeded 48 hours or used more than 60GB of memory in total.

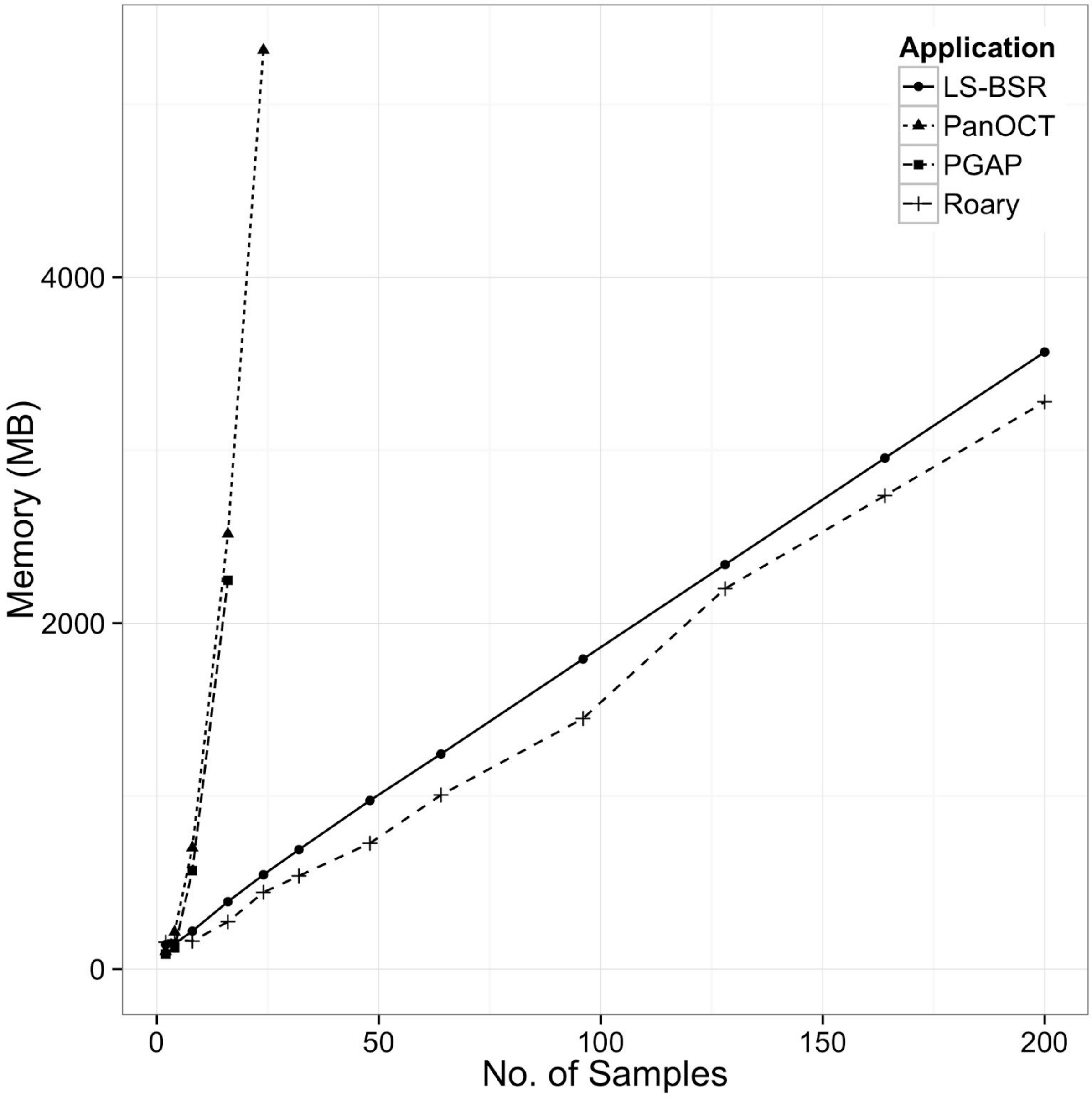

Sup. Fig. 5: Total memory (MB) used to process datasets of *S. typhi* with increasing size with 1 CPU. PanOCT and PGAP failed to work with larger datasets in under 48 hours.

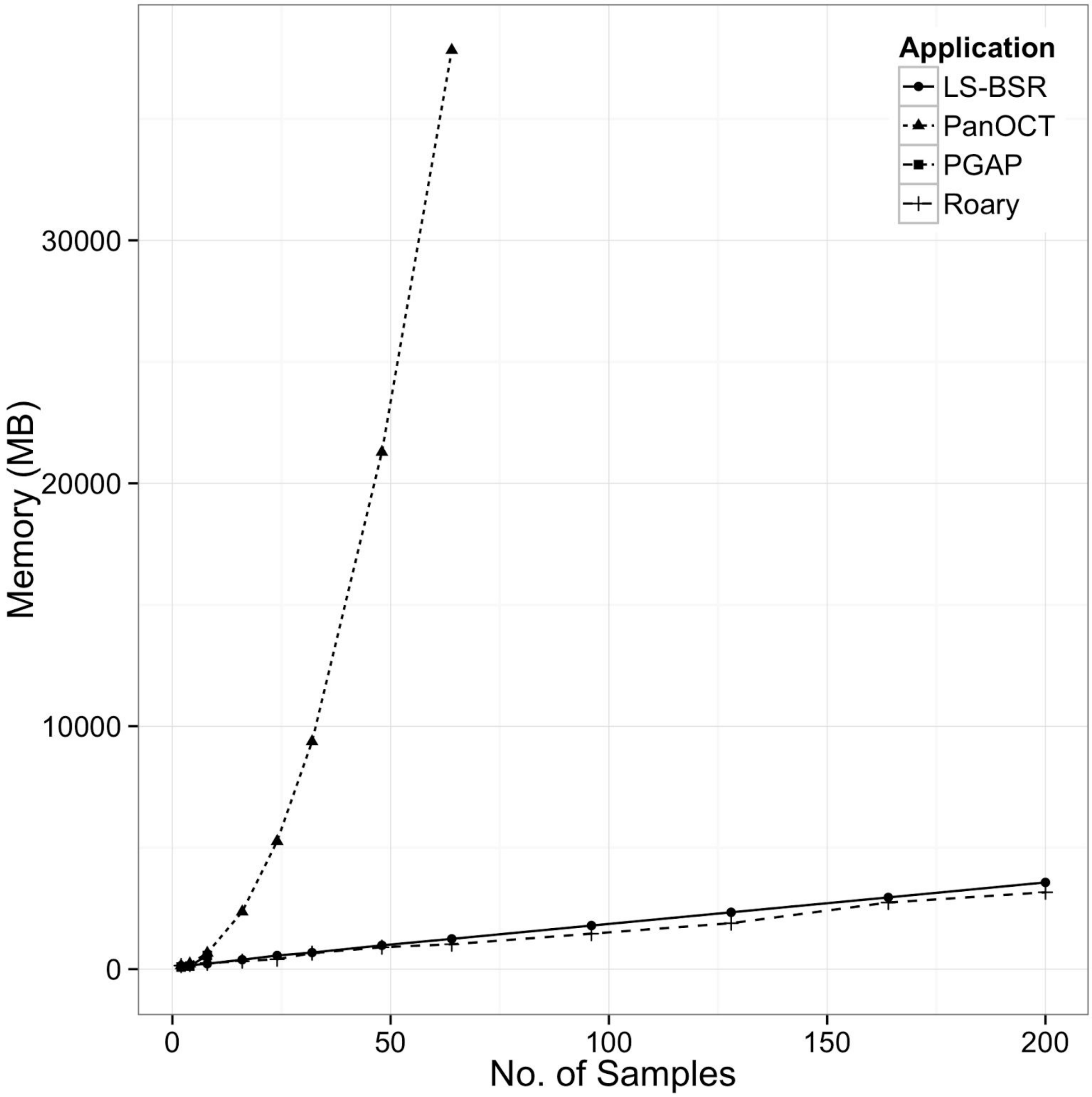

Sup. Fig. 6: Total memory (MB) used to process datasets of *S. typhi* with increasing size with 8 CPUs. PGAP failed to run with a dataset larger than 8 samples within 48 hours. PanOCT exceeded the maximum limit of 60 GB of memory while processing a dataset with 96 samples.

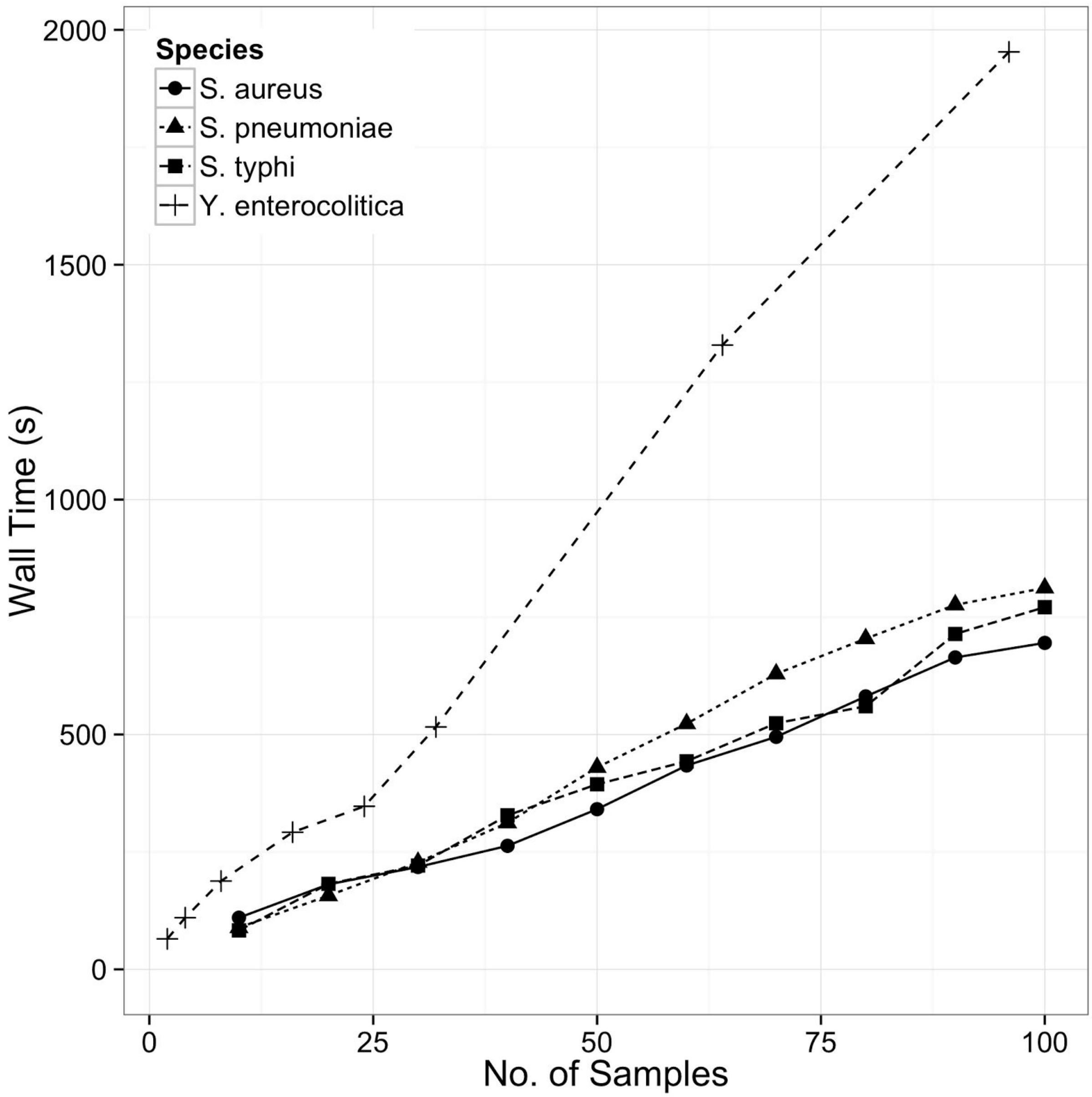
Species
S. aureus
S. pneumoniae
S. typhi
Y. enterocolitica
Wall Time (s)
2000
1500
1000
500
0
0
25
50
75
100
No. of Samples

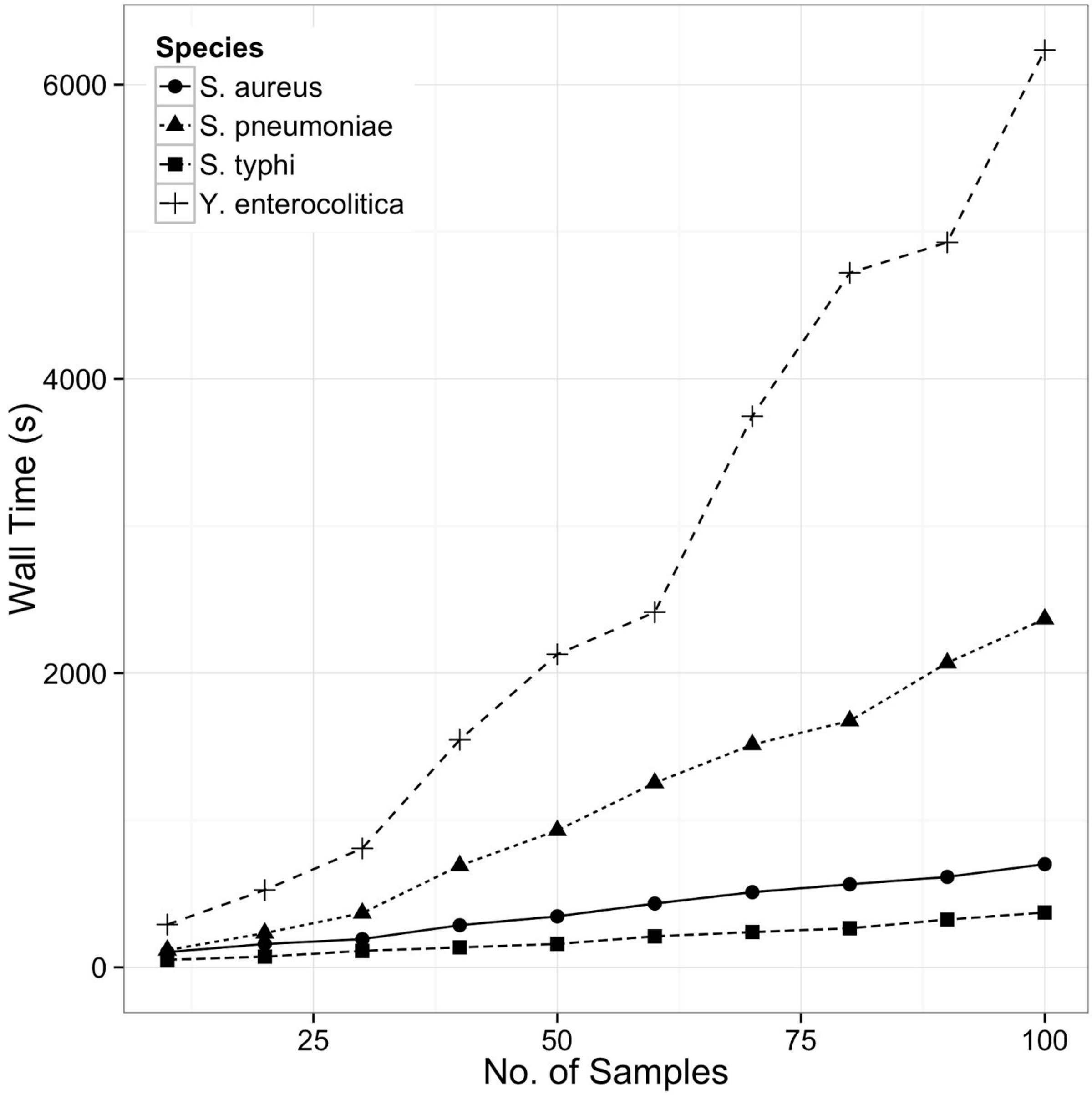

Sup. Fig. 7: Performance of Roary on a variety of organisms using 8 CPUs. Data sets of increasing complexity were chosen to demonstrate that Roary scales at a constant rate. The *S. typhi* dataset consists of a single serovar, the *S. aureus* dataset consists of 2 clonal complexes, the *S. pneumoniae* dataset was drawn from a global diversity study with multiple serotypes and the *Y. enterocolitica* dataset consists of samples multiple serotypes and multiple hosts (mammals).

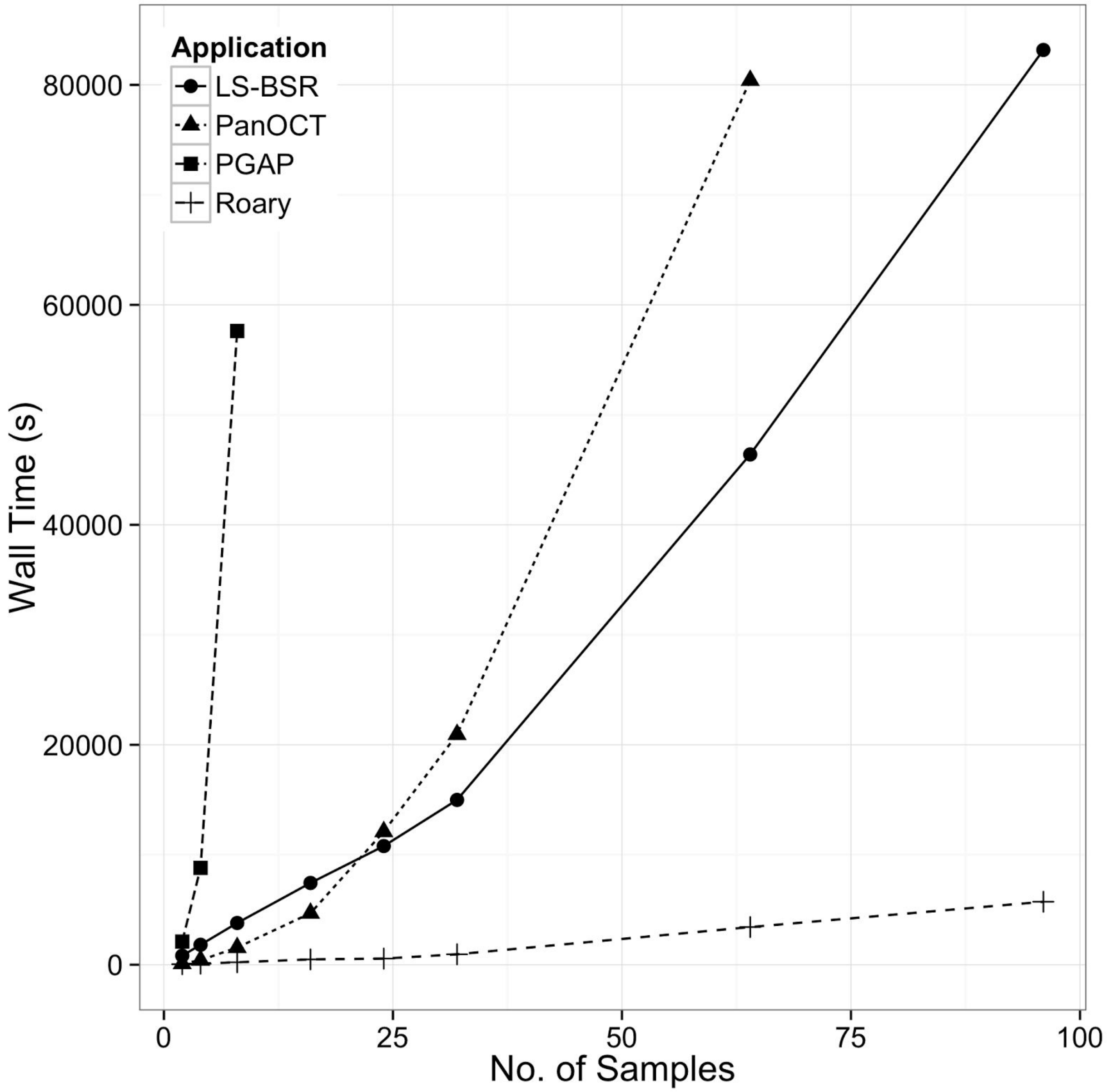

Sup. Fig. 8: The performance of multiple applications on a diverse dataset of *Y. enterocolitica* samples with increasing numbers of samples using 8 CPUs. Analysis which took over 48 hours or used more than 60 GB of RAM are not reported.

1.2 Clusters identified

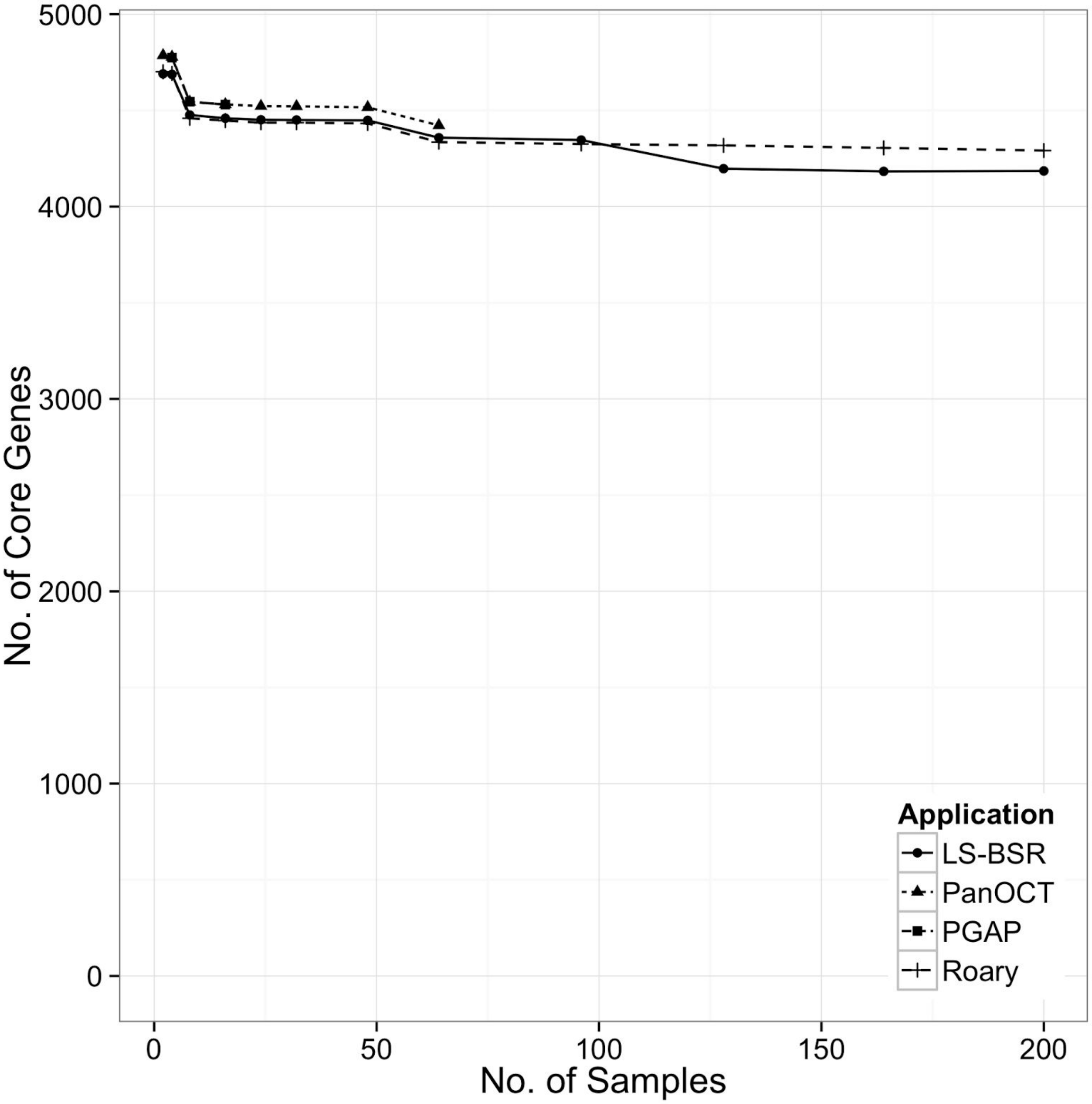

Sup. Fig. 9: The number of core genes found by each application, with increasing sizes of data sets for *S. typhi*. They all approximately predict the same number of core genes, with Roary predicting 2% less, however Roary is able to work with larger datasets. As this is a real dataset, the true values are unknown, so we do not know if Roary is underpredicting or if the other algorithms are over predicting the number of core genes. The other applications failed to produce results for the larger data sets with 8 CPUs and 60 GB of available RAM with a maximum time limit of 48 hours.

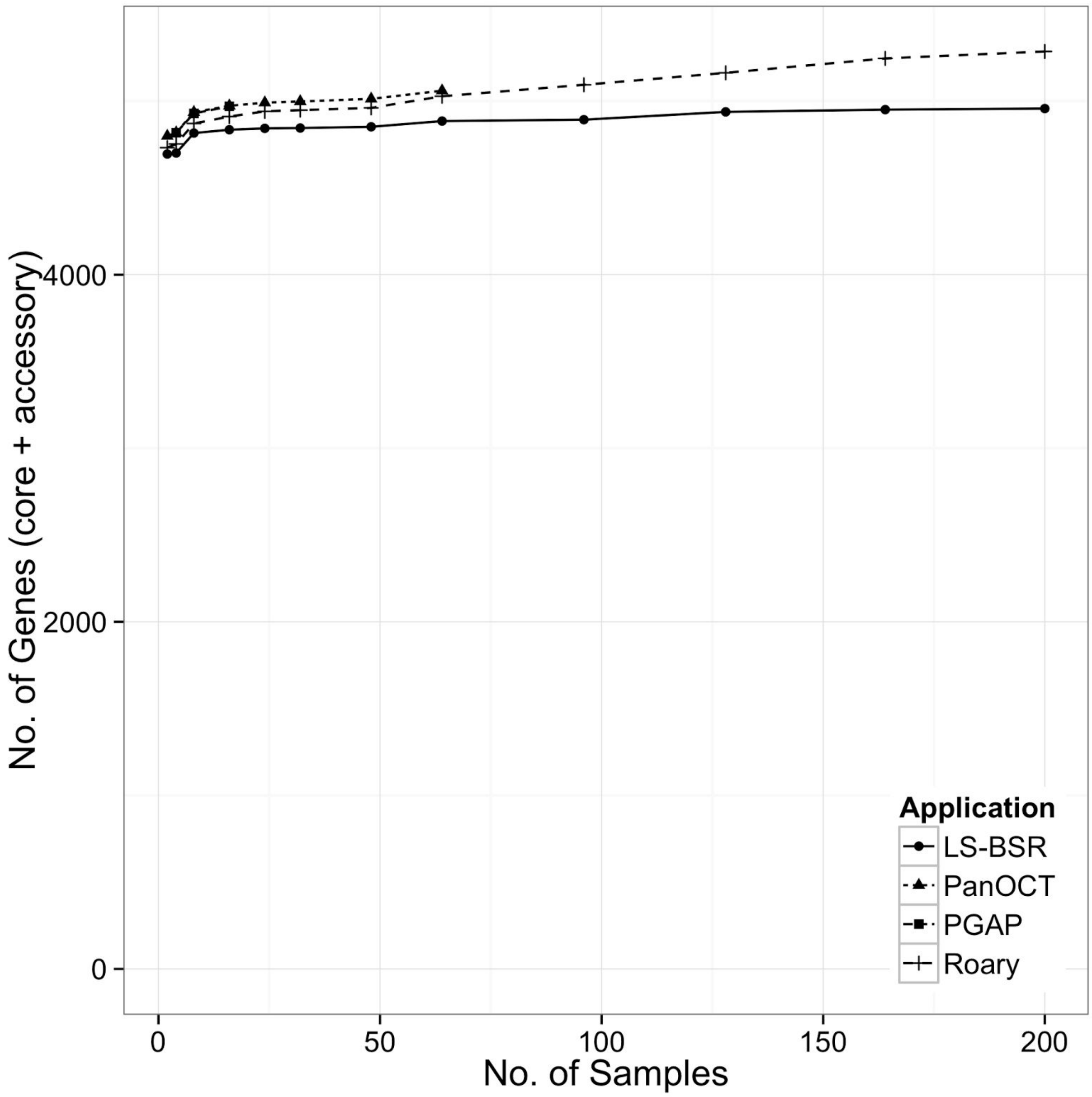

Sup. Fig. 10: Total number of genes (core + accessory) with increasing numbers of samples for *S. typhi.* PanOCT, PGAP and Roary predict values within 1% of each other.

1.3 Scaling in a multiprocessor environment

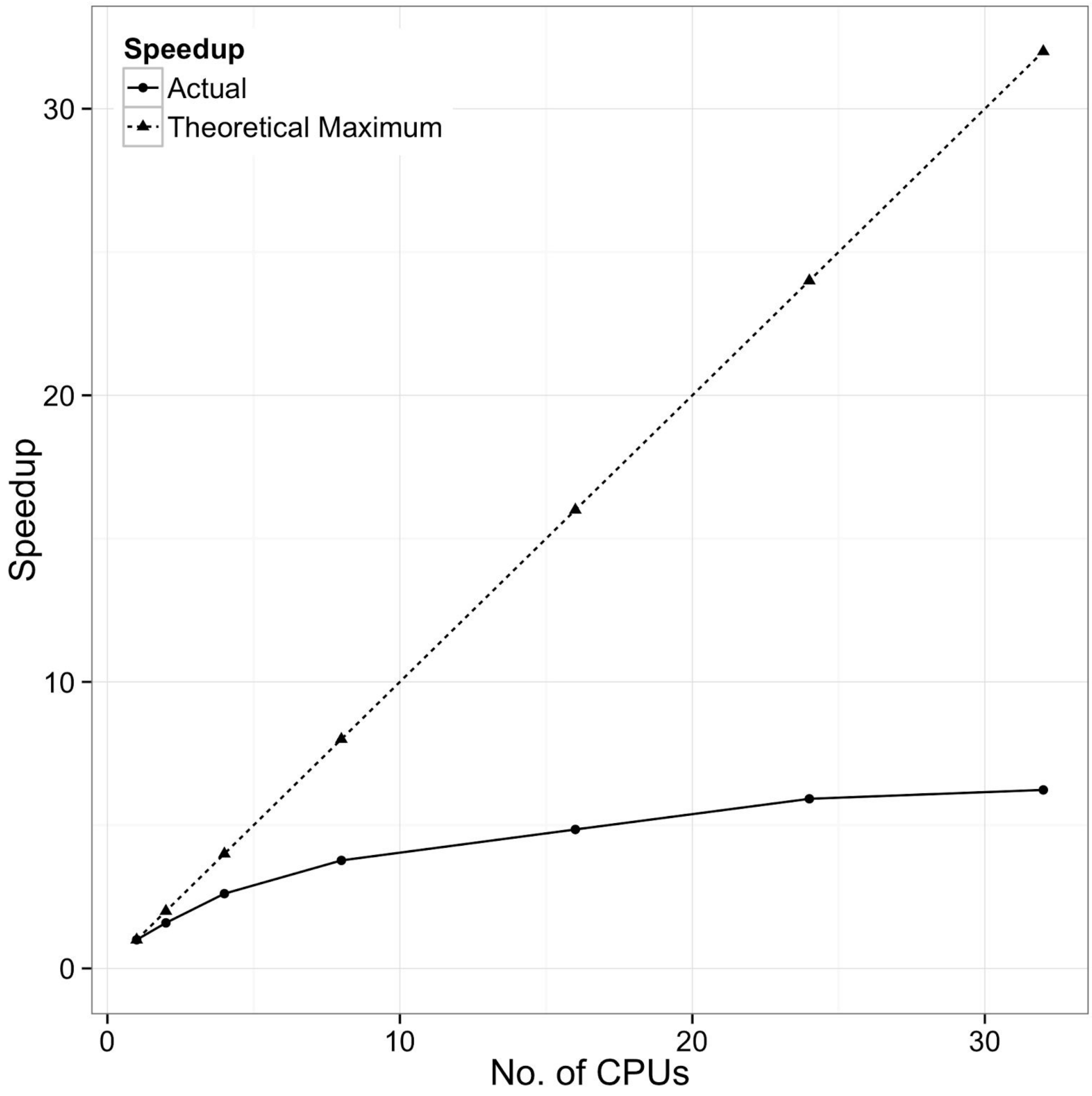

Sup. Fig. 11. The speedup achieved when using increasing numbers of CPUs. The higher the better. The dataset consisted of 100 *Y. enterocolitica* samples. GNU parallel (Tange, 2011) is used for the parallelisation.

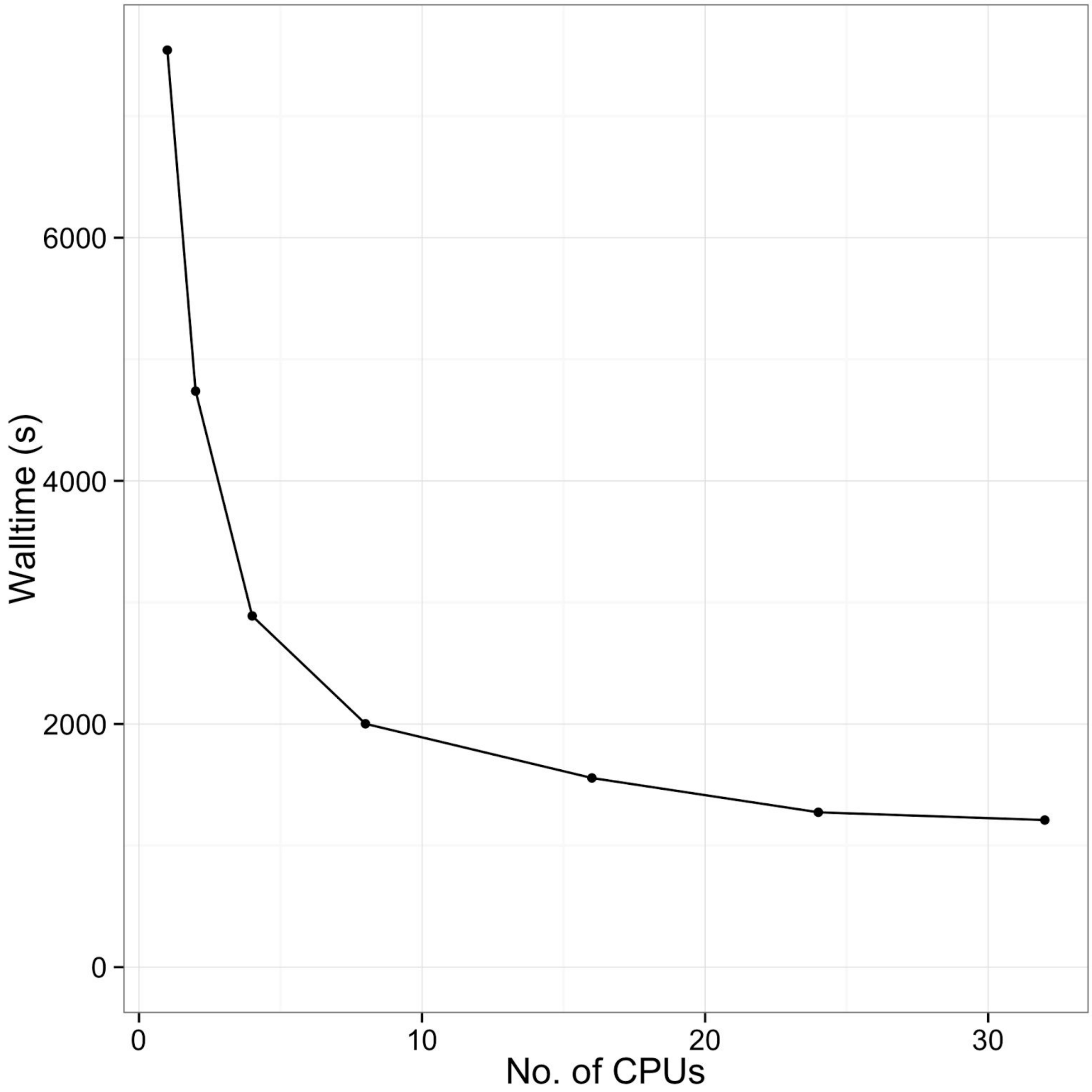

Sup. Fig. 12. The reduction in wall time achieved by increasing numbers of CPUs. This is an alternative view of the data in the previous figure. The lower the better. The dataset consisted of 100 *Y. enterocolitica* samples.

2 Method

2.1 Input

Roary accepts as input annotated *de novo* assemblies in GFF3 (Stein, 2013) format, as produced by Prokka (Seemann, 2014), where there is one file per sample. The typical input is normally fragmented draft *de novo* assemblies from short reads, or near complete draft assemblies and all of the data should be from the same species. If annotation is not available, FASTA files of amino acids can also be used as input but not all functionality is available. These are the only mandatory input files to the software.

2.2 Method description

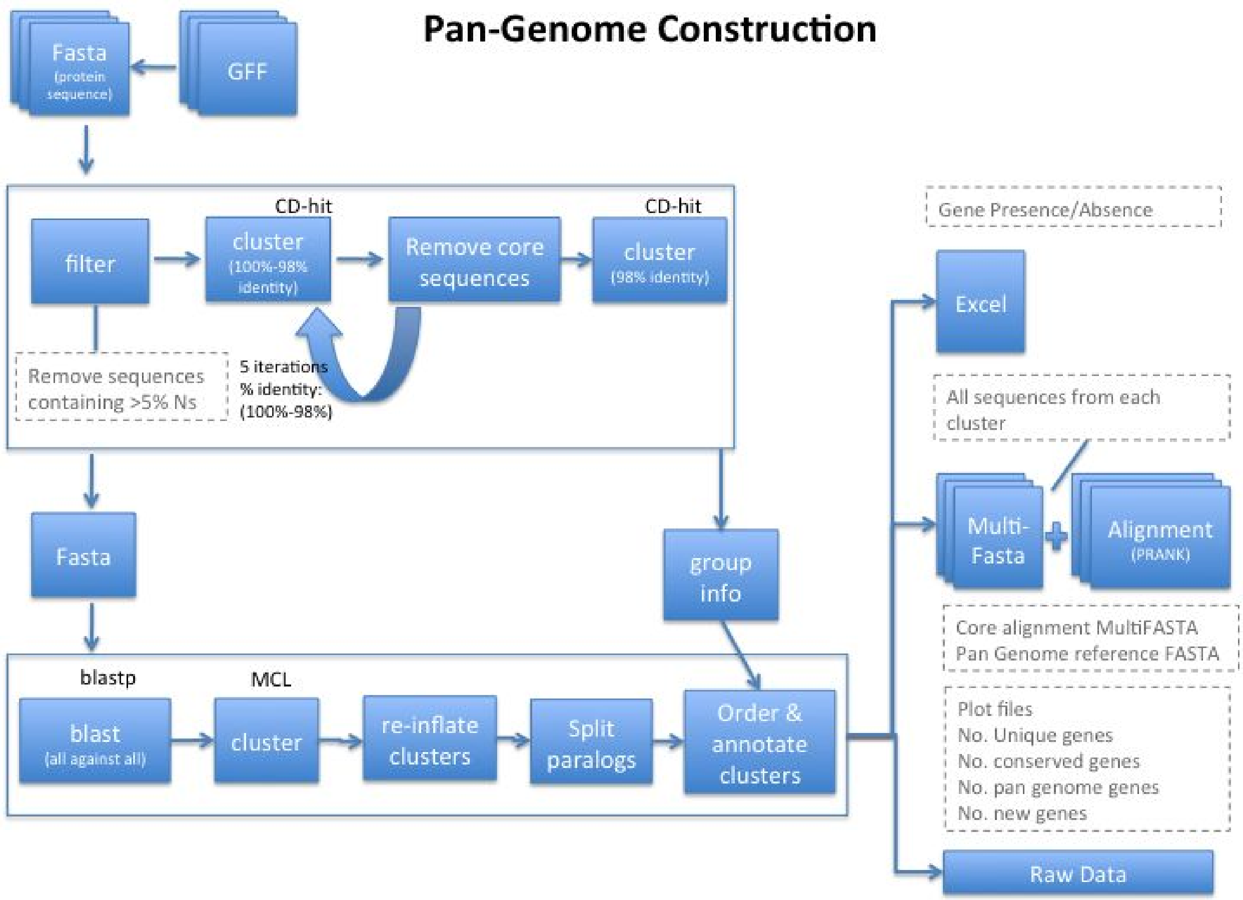

Sup. Fig. 13: A flowchart of the steps in the application.

The pipeline takes as input GFF3 files created by Prokka (Seemann, 2014) and clusters the predicted proteins to allow for the full genomic variation of the input set to be explored. The

basic method is to filter and pre-cluster the proteins, perform an all against all comparison using BLASTP, and cluster with MCL.

Predicted coding regions are extracted and converted to protein sequences. Sequences where more than 5% of nucleotides are unknown, or that are less than 120 nucleotides, are excluded from further analysis (*Common Gene Annotation Process, Broad Institute, WUGC, JCVI and Baylor*, 2011). Sequences must have a start or stop codon, and any without them are filtered out. Since the input sequences are all from the same species, there will be large numbers of near identical genes, so we use a quick clustering method to reduce the computational cost of the later all against all BLAST. This substantially reduces the running time. CD-hit (Fu, Niu, Zhu, Wu, & Li, 2012) is used to iteratively perform a first pass clustering. Beginning with a sequence identity of 100% and a matching length of 100%, the protein sequences are clustered. If a sequence is found in every isolate, it is said to be a core gene and the cluster is added to the final results. All of these sequences are then removed and not considered for analysis with BLAST. CD-hit is repeated with a lower threshold, reducing by 0.5% down to the user defined threshold (defaults to 98%), with core genes removed at each stage. One final clustering step is performed with CD-hit, with a sequence identity of 98% leaving one representative sequence for each cluster in a protein FASTA file.

A BLAST database is created from this FASTA file. Low complexity regions are first masked out with SegMasker (Camacho et al., 2008), and a protein blast database is created with makeblastdb (Camacho et al., 2008). The FASTA file is chunked up (without splitting individual sequences) and compared to the blast database to perform an all against all blast. The combined blast results are then provided as input to MCL (Enright, Van Dongen, & Ouzounis, 2002), which clusters the input sequences. It uses a normalised bit store (bit scores normalized by length of the HSP). The clusters are then re-inflated with the final CD-hit clustering results, and with the iterative CD-hit core genes. The clusters are labelled with the most commonly occurring gene names assigned to the sequences in the cluster. If there is no annotated gene name, a unique identifier is generated. The functional annotation is also recorded for each cluster.

All against all comparison with BLAST in combination with MCL clustering will produce homologous groups of genes. While useful, these groupings can often contain paralogous genes (i.e. homologs from the same genome), which could skew the results by producing falsely large and well-distributed gene groups. In theory, as pan genomes are only truly informative in closely related species, orthologous genes (homologs from different genomes) will be highly likely to also share their surrounding genes. In groups where paralogs are detected, Roary will try to split these into orthologous groups by using the conserved gene neighbourhood (CGN) of each gene.

A form of guided k-means clustering is performed using a metric for CGN sharing, assigning each of the paralogs as an initial means. Each gene in the group is assigned to the paralog with the greatest proportion of shared surrounding genes. This clustering is performed iteratively until

there are no more paralogous groups left. In cases where multiple sets of paralogs are present in the group, Roary assigns the smallest set as the means for each iteration. All results presented here uses a neighbourhood radius of 5 (5 genes before and 5 genes after).

The presence and absence of genes in the accessory genome is utilised to create a binary tree using FastTree (Price et al., 2010). This gives a rough picture of the underlying data, taking less than a second on a dataset with over 100 isolates. Whilst nowhere near as accurate as a core SNP tree, which requires substantially more computation, it does nevertheless provide useful insights and very often groups clonal isolates together. CD-hit is also used in this manner to cluster the isolates (90% identity).

The ordering of each protein, on each contig, in each *de novo* assembly, is noted. It is then used to create a graph of the ordering of the clusters, which provides a relative ordering of the clusters. Each edge is weighted by 1 divided by the number of isolates that the ordering is found in and a node corresponds to a cluster. The contribution of an isolate to the weight of an edge is based on the number of isolates in the CD-hit cluster so that low frequency genotypes are not overwhelmed by oversampled clonal isolates. Multiple graphs are created for each of the connected groups (e.g. chromosome and plasmids would be on separate graphs). To reduce the impact of spurious edges, which can be caused by mis-assemblies and mis-clustering, the graph is filtered to remove weak edges. For a given node, all edges whose weight is not within 90% of the strongest edge are removed. This greatly improves the contiguation of mobile elements, but it has the downside of disconnecting low frequency variation found within those elements. The graphs are then simplified to minimum spanning trees and traversed using depth first search. The graphs are ordered by the mean edge weight for the resulting path. The same process is repeated, but with the conserved clusters removed from the graph, to generate an overview of the accessory regions. This groups globally syntenous regions together, giving context to the genes.

Quality information can be derived about the clusters from the graphs. For example, if there are a large number of disconnected graphs, each with a small number of genes and found in low frequency in the isolates, it can indicate low copy number contamination of the isolate. A small number of these can be biologically very interesting, for example a drug resistance gene being inserted at an IS element. Also,if there are very large numbers of genes on contiguous blocks it can also indicate contamination by another organism. A further quality control step is performed where the quality of the predicted proteins is accessed based on the predicted annotation. If two genes are overlapping by more than 10% (minimum 4 nucleotides) in different reading frames it could indicate a mis-prediction. If one of the proteins is marked as a hypothetical protein, and the other has a predicted function, the hypothetical protein is flagged as potentially erroneous.

Each cluster is outputted to a separate multi-FASTA file as nucleotide sequences. They codon aligned using PRANK (Löytynoja, 2014). Genes that occur exactly once in every isolate are combined into a multiple FASTA alignment to allow for a phylogenetic tree to be constructed using the core genes.

2.3 Output

The pan genome application Roary creates multiple output files. This includes a spreadsheet detailing the presence and absence of each gene in each isolate, number of isolates a gene is found in, frequency of genes per isolate, functional annotation, QC information and sorting information. A multiple sequence file of all the nucleotide sequences for each cluster is also created and aligned using PRANK to create a multiple sequence alignment file.

Tab delimited files are created for visualizing with R (Sup. Fig. 10-13). We randomly sort the isolates files and plot what happens to the pan genome when they are added iteratively. Multiple iterations are performed because the order in which genomes are added can change the results, with the number of iterations set to the number of input files (minimum 100). This allows for bounds to be placed on the values. The number of conserved genes shows the size of the core genome (where a gene occurs at least once in every isolate) and it generally stabilises quite quickly. The total number of clusters includes the core and the accessory genome and the slope of the curve varies depending on if the pan genome is open or closed (Medini et al., 2005). The number of new, previously unseen, genes found as each isolate is added to the plot allows you to see how likely you are to find new genes as you sequence more data. Finally there is a plot for the number of unique genes overall that have been observed exactly once.

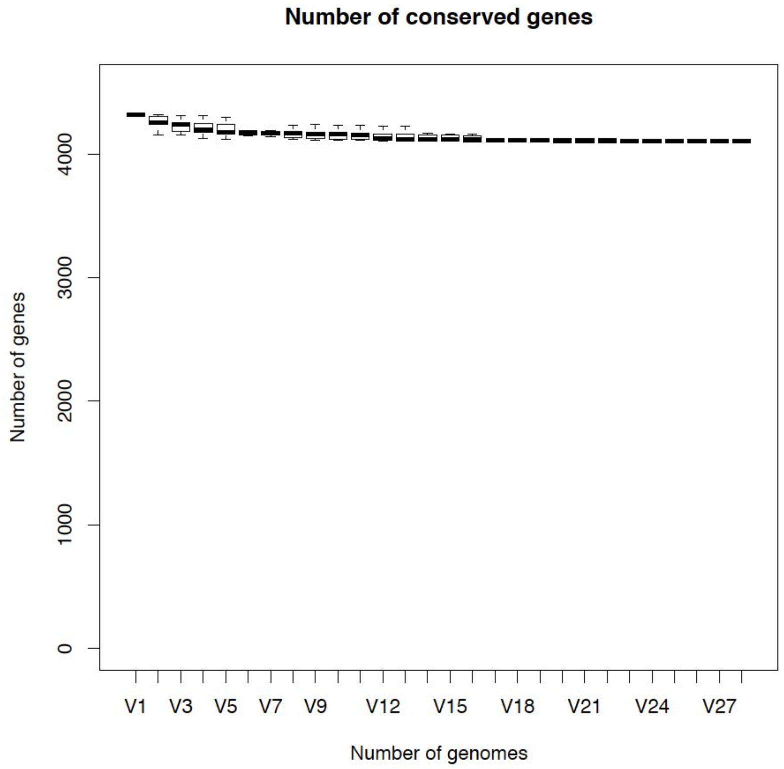

Sup. Fig. 14: Number of conserved genes.

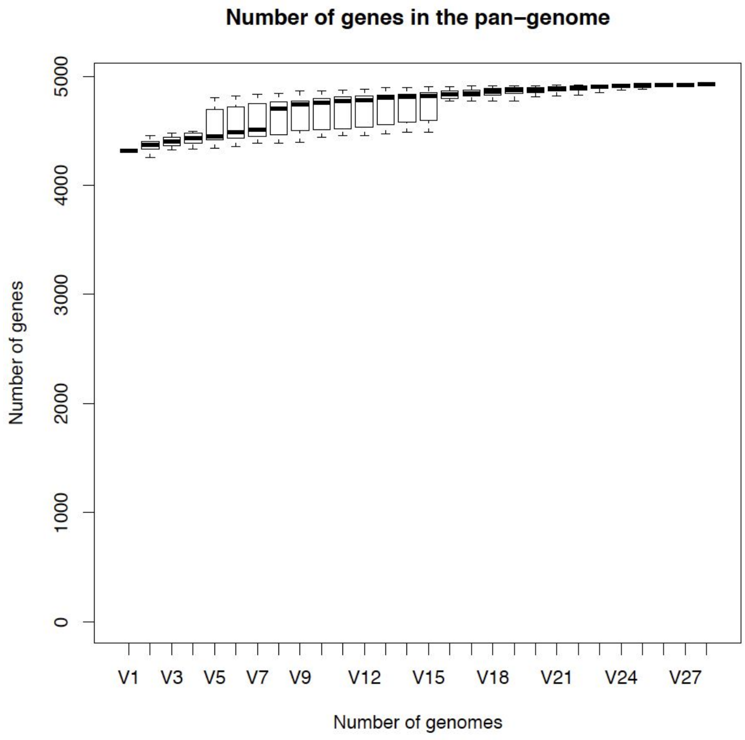

Sup. Fig. 15: Number of genes in the pan genome.

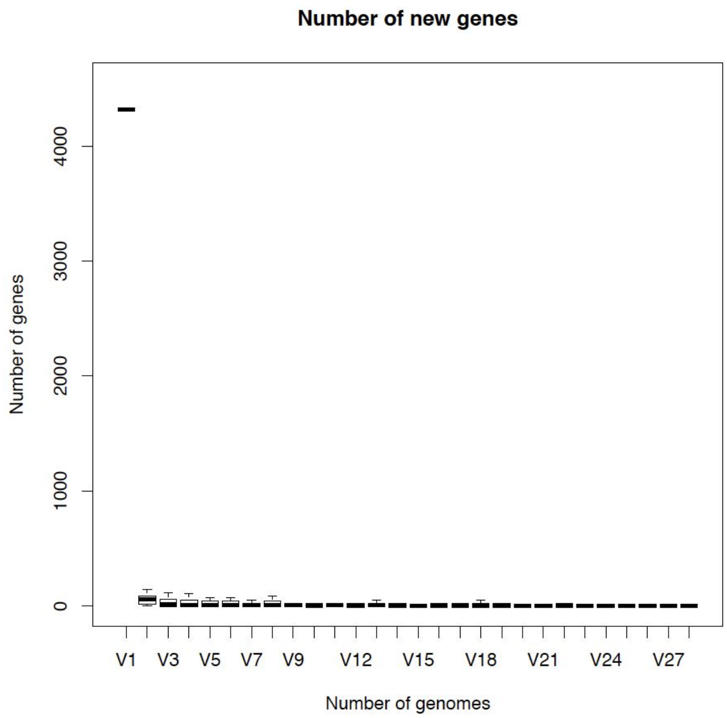

Sup. Fig. 16: Number of new genes.

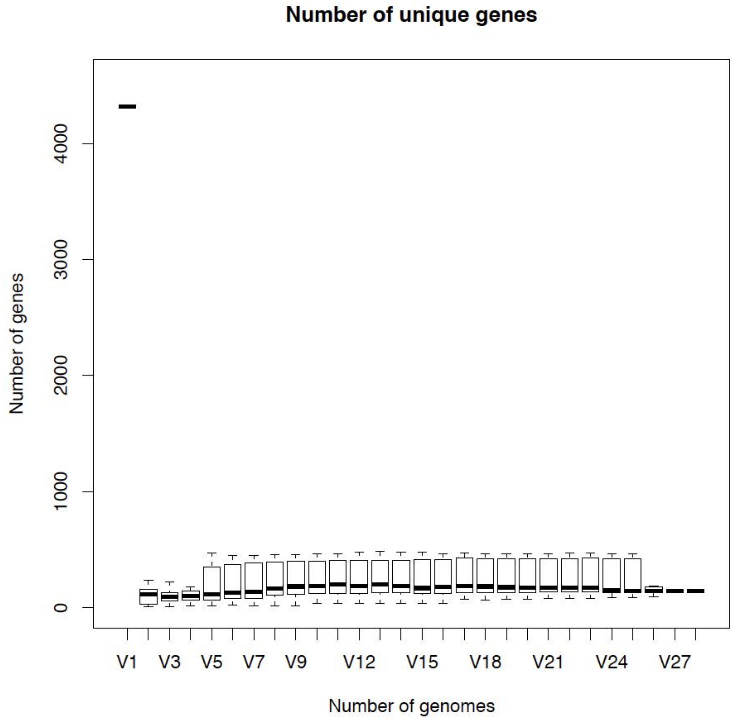

Sup. Fig. 17: Number of unique genes.

The application includes the ability to query the clusters, so that you can find out what is different about two sets of isolates and what they have in common (union, intersection, complement).

Sup. Table 1. Output files

File	Description
gene_presence_absence.csv	Spreadsheet containing statistics on each group and presence and absence of genes in each isolate
core_gene_alignment.aln	Multi-FASTA alignment of the core conserved genes
clustered_proteins	File with 1 cluster per line and sequence identifiers
*.Rtab	Tab Files for producing graphs in R
*.embl	EMBL files for visualizing presence and absence of genes
accessory_binary_genes.fa.newick	Newick tree based on presence and absence of genes in accessory genome
pan_genome_reference.fa	A FASTA file with a single nucleotide sequence for every cluster in the pan genome
pangenome_sequences	MultiFASTA alignment files for each cluster

3 Output files

3.1 Core gene alignment

If you pass in the flag '-e' a multi-FASTA file containing the aligned core genes is created. From this you can use an application like RAxML or FastTree to construct a phylogenetic tree based on SNPs in the core genes. The individual genes are codon aligned with Prank[prank]. The file is always called *core_gene_alignment.aln.*

3.2 Multifasta files of each gene

If you pass in the flags '-e --dont_delete_files' multifasta files containing nucleotide sequences will be generated for each gene. They will all reside in a sub directory called 'pan_genome_sequences'. You can use seaview to open the sequence and take a look at it. The sequences are codon aligned with Prank[prank]. Thousands of files get created so its a feature you should use with caution.

3.3 Pan Genome Reference FASTA

If you pass in the flag '-e' multifasta a FASTA file will be created called pan_genome_reference.fa. It contains a single representative nucleotide sequence for each gene/cluster in the pan genome. This can be used for aligning reads to, for passing into a reference aware assembler and for rapidly adding a new isolate to the pan genome.

4 Command line tools

4.1 Querying the pan genome

You can query the pan genome in two ways, 1.) open up the gene_presence_absence.csv spreadsheet in Excel and filter the rows and columns yourself, or 2.) use the query_pan_genome script. It takes in the groups file (clustered_proteins) and a list of the GFF files that were used to create the pan genome in the first place.

To get help run:

query_pan_genome -h

4.1.1 Difference between sets of isolates

If you have two sets of isolates, you can use this script to tell what the differences are (in terms of genes). For example, you might have a set of drug resistant isolates, and another set of susceptible isolates and you want to know are there genes how they differ. For a given pan genome, you can pass in two lists of GFF files. It will output what genes are unique to set 1, unique to set 2 and what they have in common.

query_pan_genome -a difference --input_set_one 1.gff,2.gff --input_set_two 3.gff,4.gff,5.gff -g clustered_proteins

The output files are the groups file and a spreadsheet detailing what's in common and what's unique to each set:

- set_difference_unique_set_one_statistics.csv
- set_difference_unique_set_one
- set_difference_unique_set_two_statistics.csv
- set_difference_unique_set_two
- set_difference_common_set_statistics.csv
- set_difference_common_set

4.1.2 Create multi-FASTA files for a list of genes

This action will create a multi-FASTA file of protein sequences for the list of genes passed in. The data is extracted directly from the GFF files and you can choose nucleotide sequences or amino acid sequences. The sequences are not aligned.

*query_pan_genome -a gene_multifasta -g clustered_proteins -n gryA,mecA,abc *.gff*

4.1.3 Union (pan genome)

Get the union of the genes for the GFF files passed in. This will give you all of the genes that are found in any of the isolates used to create the pan genome. This is particularly useful if you have created a pan genome, and you spot a clade of interest. It allows you to then zoom in on the clade and find out what genes they have in common.

*query_pan_genome -a union -g clustered_proteins *.gff*

To get the list of genes found in a subset of GFF files:

query_pan_genome -a union -g clustered_proteins file1.gff file2.gff file3.gff

4.1.4 Intersection (core genes)

Get the intersection of the genes for the GFF files passed in. This will give you all the genes that are found in all isolates (e.g. the core genes) for the given list of GFF files. This allows you to find out what the core genes are for a subset of isolates.

*query_pan_genome -a intersection -g clustered_proteins *.gff*

To get the core genes for a subset of GFF files:

query_pan_genome -a intersection -g clustered_proteins file1.gff file2.gff file3.gff

4.1.5 Complement (accessory genes)

Get the complement, otherwise known as the accessory genes, of a subset of isolates. It is the Union minus the Intersection.

*query_pan_genome -a complement -g clustered_proteins *.gff*

To get the accessory genes for a subset of GFF files:

query_pan_genome -a intersection -g clustered_proteins file1.gff file2.gff file3.gff

5 References

Camacho, C., Madden, T., Ma, N., Tao, T., Agarwala, R., & Morgulis, A. (2008). *BLAST Command Line Applications User Manual*. Bethesda (MD). Retrieved from http://www.ncbi.nlm.nih.gov/books/NBK1763/

Chewapreecha, C., Harris, S. R., Croucher, N. J., Turner, C., Marttinen, P., Cheng, L., … others. (2014). Dense genomic sampling identifies highways of pneumococcal recombination. *Nature Genetics*, *46*(3), 305–309.

Common Gene Annotation Process, Broad Institute, WUGC, JCVI and Baylor. (2011). Retrieved from http://hmpdacc.org/doc/CommonGeneAnnotation_SOP.pdf

Enright, A. J., Van Dongen, S., & Ouzounis, C. A. (2002). An efficient algorithm for large-scale detection of protein families. *Nucleic Acids Research* , *30* (7), 1575–1584. doi:10.1093/nar/30.7.1575

Fouts, D. E., Brinkac, L., Beck, E., Inman, J., & Sutton, G. (2012). PanOCT: automated clustering of orthologs using conserved gene neighborhood for pan-genomic analysis of bacterial strains and closely related species. *Nucleic Acids Research* , *40* (22), e172–e172. doi:10.1093/nar/gks757

Fu, L., Niu, B., Zhu, Z., Wu, S., & Li, W. (2012, December). CD-HIT: accelerated for clustering the next-generation sequencing data. *Bioinformatics*. doi:10.1093/bioinformatics/bts565

Löytynoja, Ari. Phylogeny-aware alignment with PRANK. Methods in molecular biology (Clifton, N.J.) 1079:155-170 (2014).

Medini, D., Donati, C., Tettelin, H., Masignani, V., & Rappuoli, R. (2005). The microbial pan-genome. *Current Opinion in Genetics & Development*, *15*(6), 589–594.

Nguyen, N., Hickey, G., Zerbino, D. R., Raney, B., Earl, D., Armstrong, J., … Paten, B. (2015). Building a Pan-Genome Reference for a Population. *Journal of Computational Biology : A Journal of qComputational Molecular Cell Biology*. doi:10.1089/cmb.2014.0146

Price, M.N., Dehal, P.S., Arkin, A.P. (2010). FastTree 2 – Approximately Maximum-Likelihood Trees for Large Alignments. *PLoS ONE* 5(3): e9490. doi: 10.1371/journal.pone.0009490

Reuter, S., Connor, T. R., Barquist, L., Walker, D., Feltwell, T., Harris, S. R., … Thomson, N. R. (2014). Parallel independent evolution of pathogenicity within the genus Yersinia. *Proceedings of the National Academy of Sciences of the United States of America*, *111*, 6768–73. doi:10.1073/pnas.1317161111

Seemann, T. (2014). Prokka: rapid prokaryotic genome annotation. *Bioinformatics (Oxford, England)*, *30*(14), 2068–2069. doi:10.1093/bioinformatics/btu153

Stein, L. (2013). Generic Feature Format Version 3 (GFF3). Retrieved from http://www.sequenceontology.org/gff3.shtml

Tange O. (2011) GNU Parallel - The Command-Line Power Tool. ;login: The USENIX Magazine, **36**, 42-47.

Vernikos, G., Medini, D., Riley, D. R., & Tettelin, H. (2014). Ten years of pan-genome analyses. *Current Opinion in Microbiology*, *23C*, 148–154. doi:10.1016/j.mib.2014.11.016

Zhao, Y., Jia, X., Yang, J., Ling, Y., Zhang, Z., Yu, J., … Xiao, J. (2014). PanGP: A tool for quickly analyzing bacterial pan-genome profile. *Bioinformatics* , *30* (9), 1297–1299. doi:10.1093/bioinformatics/btu017

Zhao, Y., Wu, J., Yang, J., Sun, S., Xiao, J., & Yu, J. (2012). PGAP: pan-genomes analysis pipeline. *Bioinformatics* , *28* (3), 416–418. doi:10.1093/bioinformatics/btr655
